# Patterns of Thyroid Cancer Mortality and Incidence in Saudi Arabia: A 30-Year Study

**DOI:** 10.3390/diagnostics12112716

**Published:** 2022-11-07

**Authors:** Arwa F. Flemban, Saeed Kabrah, Hanaa Alahmadi, Raghad K. Alqurashi, Anwar S. Turaes, Ruba Almaghrabi, Samah Al Harbi, Asim A. Khogeer

**Affiliations:** 1 Pathology Department, Faculty of Medicine, Umm Al-Qura University, P.O. Box 50199, Makkah 21955, Saudi Arabia; 2 Laboratory Medicine Department, Faculty of Applied Medical Sciences, Umm Al-Qura University, P.O. Box 50199, Makkah 21955, Saudi Arabia; 3 Faculty of Medicine, Umm Al-Qura University, P.O. Box 50199, Makkah 21955, Saudi Arabia; 4 Laboratory Medicine Department, Faculty of Applied Medical Sciences, Albaha University, Albaha 65431, Saudi Arabia; 5 Physiology Department, Faculty of Medicine, Umm Al-Qura University, P.O. Box 50199, Makkah 21955, Saudi Arabia; 6 Research Department, The Strategic Planning, General Directorate of Health Affairs Makkah Region, Ministry of Health, Makkah 24354, Saudi Arabia; 7 Medical Genetics Unit, Maternity & Children Hospital, Makkah Healthcare Cluster, Ministry of Health, Makkah 24354, Saudi Arabia

**Keywords:** thyroid cancer, Saudi Arabia, prevalence, incidence, death, retrospective study

## Abstract

Thyroid cancer is the most prevalent endocrine cancer among the female population in the Kingdom of Saudi Arabia (KSA) and the ninth most common in the male population in Saudi Arabia. Over the past years, an increasing incidence of thyroid cancer has been reported in Saudi Arabia. However, the etiology of thyroid cancer is still not clear. Therefore, this study aimed to estimate thyroid cancer incidence and mortality trends in Saudi Arabia from 1990 to 2019. The current study utilized the Global Burden of Disease and the Institute for Health Metrics and Evaluation databases to extract prevalence data of thyroid cancer in Saudi Arabia from 1990 to 2019. Moreover, the current project utilizes Global Burden of Disease (GBD) web-based tools to visualize these data. In total, 23,846 cases (17,220 females and 6626 males) were diagnosed with thyroid cancer in Saudi Arabia from 1990 to 2019. The incidence is higher in females than in males. Over these 30 years, women’s incidence steadily increased by 15-fold versus a 22-fold increase in men. Moreover, there were 2056 deaths in total caused by thyroid cancer in KSA. The mortality rate in women steadily increased by threefold in the same period. However, the increase in mortality was higher in males (sixfold). A high percentage of YLLs was observed in males, with around 24.8% ranging from 30 to 34 and 40 to 45 years. Thyroid cancer incidence rates have increased exponentially between 1990 and 2019. The expansion of the incidence of thyroid cancer in Saudi Arabia could be due to the increased development in detection and diagnosis. The current study provided evidence of the need to increase awareness and diagnosis in the male population.

## 1. Introduction

Thyroid cancer is the most prevalent endocrine malignancy [1]. It is the second-most- common malignancy in women after breast cancer and the ninth-most-common cause of all cancers in women worldwide [2]. Its incidence has increased over the last few decades; however, mortality rates have steadily declined. Even so, the death rate from thyroid cancer has increased faster than that of any other malignancy in both men and women. Increased risk factors include genetic factors, age, environmental and lifestyle changes, radiation exposure, smoking, alcohol intake, pregnancy, oral contraceptives, and high levels of thyrotropin [3].

During the last three decades, Saudi Arabia has experienced important demographic, economic, and cultural changes [4]. These changes have impacted clinical practice, pathological classification, and management tools with respect to health conditions, including thyroid cancer [5]. The Saudi National Cancer Registry has reported a significant increase in the incidence of thyroid cancer in both males and females [4,6]. A material understanding of thyroid cancer trends is critical in the planning of national cancer prevention and control strategies. In forming a national Saudi health policy, an awareness of age- and sex-specific mortality rates and trends is essential for all health conditions. In this study, we sought to estimate thyroid cancer incidence and mortality trends in Saudi Arabia in the years 1990–2019.

## 2. Materials and Methods

### 2.1. Data Collection

The current study utilized data from the Saudi Arabian National Cancer Registry and applied the general method described in the previously published Global Burden of Disease (GBD) study [7]. We accessed the Institute for Health Metrics and Evaluation database (IHME, 2017) to extract prevalence data for thyroid cancer in Saudi Arabia from 1990 to 2019. We specified database criteria as follows: “Saudi Arabia” for the location; “B.1.23 Thyroid Cancer” for the cause; and “death, incidence, and prevalence” for measures. Our analysis also involved variables related to the thyroid cancer mortality rate per 100,000 population in Saudi Arabia measured in years of life lost (YLLs), years lived with disability (YLDs), and disability-adjusted life years (DALYs). In line with the GBD method, we calculated YLLs by subtracting the age of death of individuals with the disease from the longest life expectancy of individuals of the same age. We calculated YLDs by multiplying the prevalence of thyroid cancer by the disability weight of the disease, which reflects the severity of the condition.

We adopted our definitions of localized, regional, and distant cancers from the 2017 Saudi National Cancer registry report, which used the *International Classification of Diseases for Oncology* (3rd edition) (ICD-O-3) published by the World Health Organization (WHO) in 2000. We carried out staging according to SEER coding (Summary Stage Manual 2000). According to the SEER database, localized thyroid cancer is a tumor without signs that cancer has spread outside the thyroid. When the tumor spreads outside the thyroid to nearby structures and lymph nodes, it is classified as regional. Finally, a distant cancer involves the spread of tumors to distant parts of the body, such as the bones [8].

### 2.2. Data Analysis and Visualization

We completed our data analysis on 1 October 2021. We used Excel to carry out descriptive analysis of GBD prevalence data by gender and year. We used the web-based Global Health Data Exchange (GDHx) tool to visualize thyroid cancer cause-of-death and age-distribution patterns in Saudi Arabia. We presented our data using Graphpad Prism version 9.0.0 (GraphPad Software Inc., California, United States). We determined changes for each parameter by comparing corresponding data for the years 1990 and 2019. We expressed our data as frequency, mean, and 95% confidence intervals (95%CI). Finally, we analyzed our thyroid cancer data in terms of 5-year periods within the 1990–2019 study period as well as in terms of 19 age groups (ranging from 0 to 95+ years).

## 3. Results

### 3.1. Increased Incidence of Thyroid Cancer in Saudi Arabia over the 30-Year Study Period

A total of 23,846 new cases of thyroid cancer (17,220 females and 6626 males) were diagnosed in Saudi Arabia during the period from 1990 to 2019, according to the IHME database. Over this 30-year period, we found a steady increase in thyroid cancer cases in the Saudi population of both genders. However, we found a greater rate of increase in males (104 females in 1990, 1551 in 2019; 32 males in 1990, 708 in 2019). On average, the incidence of thyroid cancer increased by a factor of 1.1 annually in both females and males. Overall, between 1990 and 2019, thyroid cancer cases rose in the female and male populations by 15- and 22-fold, respectively.

**Figure 1:**
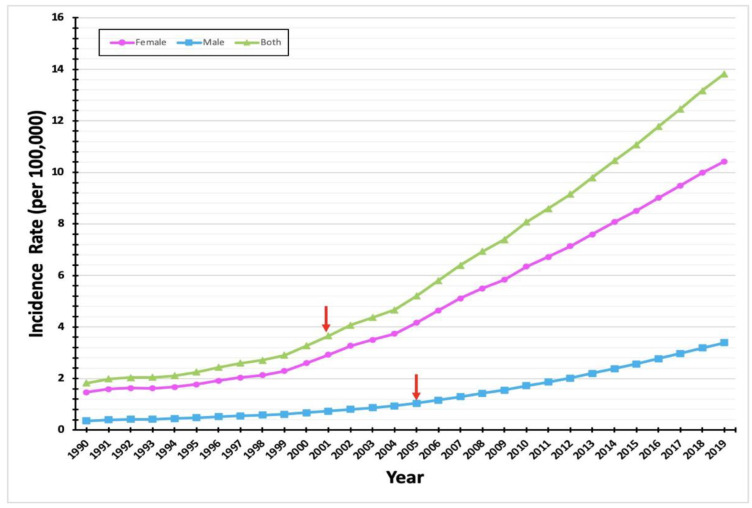
Increased incidence of thyroid cancer in Saudi Arabia over the 30-year study period. The line chart illustrates the rate of cases diagnosed with thyroid cancer in Saudi Arabia from 1990 to 2019. The green line represents the total number of cases per year (male and female cases); the pink line represents the number of female cases per year; and the blue line represents the number of male cases per year. The red arrow highlights the point in which there were significant change in the increase of incidence rate in 2000 for female (left) and in 2005 for male (right).

We found that a sharp increase in female cases started in 2000 and reached its peak in 2019; a similar increase in cases among males began in 2005 (highlighted by the red arrow to the right in Figure 1). In 1990, female and male thyroid cancer cases represented 76.5% and 23.5%, respectively, of all thyroid cancer cases in Saudi Arabia. In 2019, the corresponding figures were 68.7% for females and 31.3% for males (Figure 1).

### 3.2. Thyroid Cancer Mortality Increased in Saudi Arabia in the Past 30 Years, with a Growing Proportion of Deaths among Males

**Figure 2:**
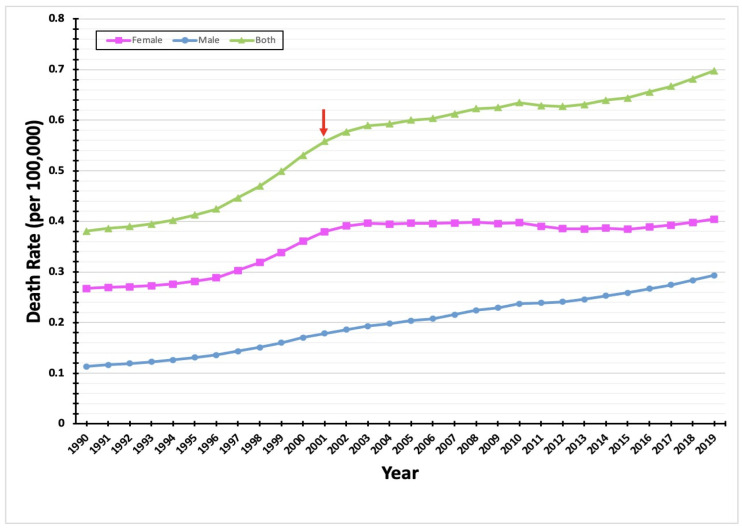
An increase in the number of deaths caused by thyroid cancer over the 30-year study period. The line chart illustrates the number of deaths caused by thyroid cancer in Saudi Arabia from 1990 to 2019. The green line represents the total number of deaths caused by thyroid cancer per year (male and female cases); the pink line represents the number of deaths caused by thyroid cancer in females per year; and the blue line represents the number of deaths caused by thyroid cancer in males per year. The red arrow highlights the point in which there were significant change in the increase of death rate in 2000.

During the period 1990–2019, there were 2056 deaths caused by thyroid cancer in Saudi Arabia. Over the course of this period, annual death rates rose dramatically, from less than 30 deaths in 1990 to more than 120 deaths in 2019, a nearly fourfold increase. In women, thyroid cancer mortality increased by a factor of three (from 19 to 60 deaths) between 1990 and 2019, with a steady increase from 1990 until 2000 (highlighted by the red arrow in Figure 2) and a more dramatic rise in subsequent years. In men, mortality increased by a factor of six (from 10 to 61 deaths) between 1990 and 2019. By the end of this period, the number of male deaths from thyroid cancer was almost the same as for females. Among female thyroid cancer cases, the percentage of patients who died declined from 18% in 1990 to less than 4% in 2019. We also found a corresponding decline in deaths among male cases, from 31% in 1990 to less than 9% in 2019. Overall, 65.5% of thyroid cancer deaths in 1990 were women, but less than 50% were women in 2019. Correspondingly, men made up only 34.5% of deaths in 1990, but more than 50% of deaths in 2019 (Figure 2).

The female mortality due to thyroid cancer from overall deaths by the same cancer dropped from 65.5% in 1990 to almost 50% in 2019. In addition, the male mortality of the same cancer increased from 34.5% in 1990 to more than 50% in 2019 (Figure 2).

### 3.3. Thyroid Cancer Mortality Increased in Saudi Arabia in the Past 30 Years, with More Male Deaths

**Figure 3:**
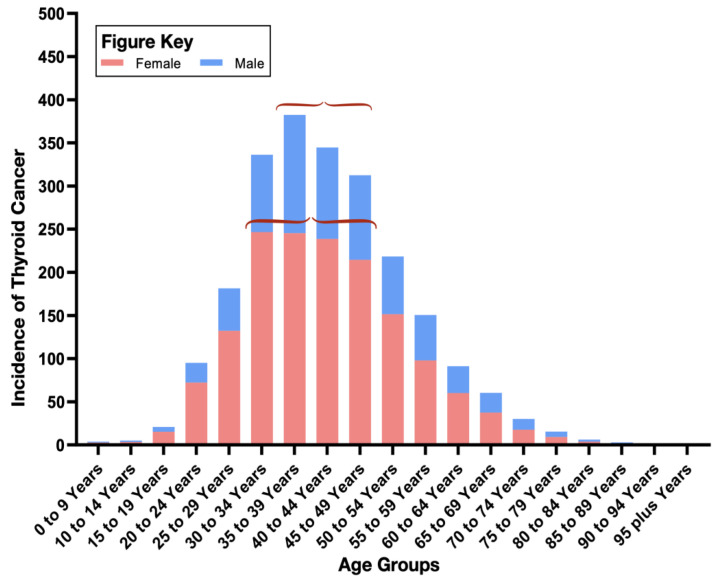
Age-group distribution of thyroid cancer incidence in 2019. Stacked columns show thyroid cancer incidence among males (blue) and females (pink) in Saudi Arabia in 2019. Red brackets highlight the highest-incidence age group.

In terms of gender, we found total deaths from thyroid cancer were almost perfectly balanced between males and females by 2019. We then turned our attention to the age distribution of thyroid cancer incidence, in order to see if we could to identify any age groups at high risk from the disease. Our data analysis revealed that 60.9% of thyroid cancer cases among females were in women between 30 and 49 years old. Among males, we found a peak incidence of 48.16% in men aged between 35 and 49 years, a slightly older age group (Figure 3).

### 3.4. Changes in Thyroid Cancer Prevalence, Incidence, Death, YLLs, YLDs, and DALYs in Females and Males over the 30-Year Study Period

Table 1 shows that, during 2019, the prevalence of thyroid cancer was higher in females (69.3%) compared with males (30.7%). In the same year, the total number of new thyroid cancer cases reported was 2259, with the incidence again higher in females (68.7%) than in males (31.3%). Despite a consistent predominance of female cases during 1990–2019, deaths among males have progressively increased over the same period, such that men now comprise a majority of thyroid cancer deaths (50.4%). Despite the growth in thyroid cancer prevalence, incidence, and mortality in both genders between 1990 and 2019, prevalence among males has increased by 25.1%, incidence has risen by 20.9%, and mortality has increased by 5%.

In terms of YLLs, YLDs, and DALYs, we found significant percentage changes over the 1990–2019 period (Table 1). Our data analysis revealed that females had nearly the same YLLs as males (50.1 and 49.8%, respectively), while both YLDs and DALYs were higher in females (68.2% and 53.9%) than in males (31.8% and 46%). The most surprising aspect of the data was found when we compared the 1990 figures for YLLs, YLDs, and DALYs with the corresponding data for 2019. We found that, among males, YLLs increased by 5.7%, YLDs increased by 22.4%, and DALYs increased by 6.5%. These results suggest that Saudi Arabian men have a greater risk of thyroid cancer incidence and mortality than males in the overall global population.

### 3.5. YLDs and YLLs of Thyroid Cancer—Age and Gender Patterns

**Figure 4:**
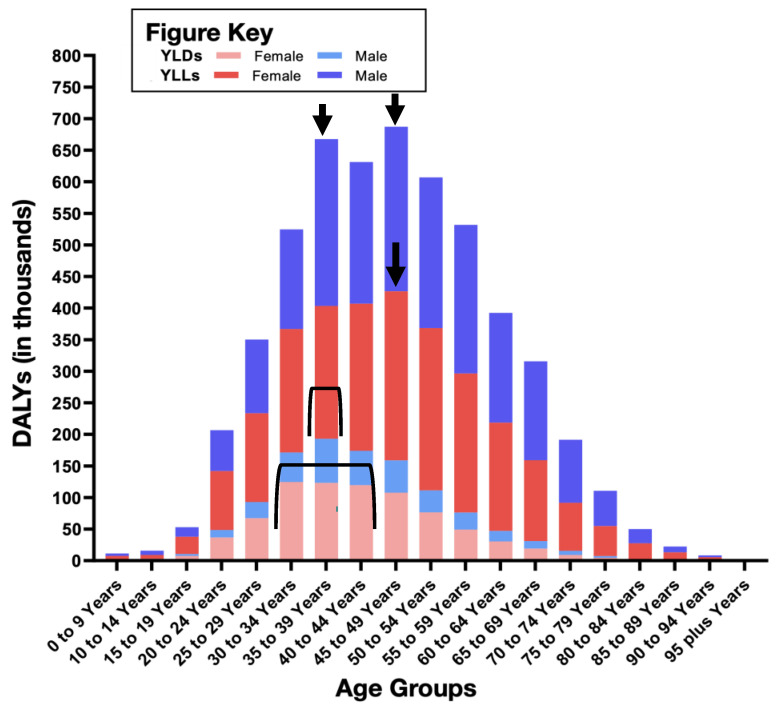
Age-group distribution of thyroid cancer YLDs and YLLs in 2019. Stacked columns show thyroid cancer incidence among males (blue) and females (pink) in Saudi Arabia in 2019. Black brackets highlight the highest-mortality age group. The black arrows highlight the age groups with highest YLLs. The black brackets highlight the age groups with the highest YLDs.

We calculated those deaths caused by thyroid cancer represented the loss of 66,757 years of life due to mortality over the 30-year study period. We analyzed the age distribution of YLDs and YLLs in 2019 to identify age groups at higher risk of thyroid cancer disability and lost years of life. In females, we found that 47% of years lived with thyroid cancer disability involved women aged between 30 and 44 years. Among males, we found that approximately 19.2% of years lived with a thyroid cancer disability involved men aged from 35 to 39 years. In females, we found a peak figure for lives lost due to thyroid cancer of around 12.6% in women aged between 45 and 49 years. In males, 24.8% of lives lost involved men aged from 30 to 34 and 45 to 49 years. These results indicate that women can live longer with thyroid cancer, while men are more likely to lose their lives after being diagnosed. The data also indicate the severe effect of thyroid cancer on several male age groups (Figure 4).

### 3.6. Morphological-Type Distribution of Thyroid Cancer among the Saudi Population

Based on the previous analysis, we sought to identify the most common morphological type of thyroid cancer in males and females. Table 2 shows that, in both genders, the most common type of thyroid cancer was papillary adenocarcinoma NOS (54.7% in males and 53.4% in females), followed by papillary carcinoma follicular variant and papillary microcarcinoma. The most aggressive types, such as medullary carcinoma and NOS, were more present in males by 3%.

### 3.7. Stage Distribution of Thyroid Cancer among the Saudi Population

**Figure 5:**
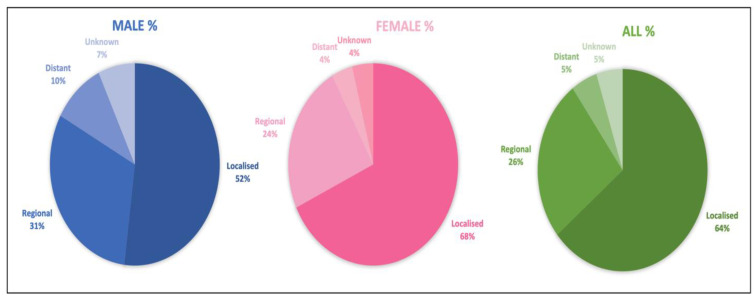
Stage distribution of thyroid cancer among the Saudi population, 2017. Data adapted from the Saudi Arabia National Cancer Registry 2017.

Figure 5 shows our analysis of thyroid cancer staging in both genders. Among females, 68% of cases were localized tumors, while 32% involved metastasis (24% regional, 4% distant, and 4% unknown). Among males, 52% of cases were localized, while 48% involved metastasis (31% regional, 10% distant, and 5% unknown). These findings help to explain the higher mortality in men of younger ages. Typically, males present with a more aggressive stage or type of thyroid cancer at the time of diagnosis, and this results in a higher figure of YLLs.

## 4. Discussion

Globally, in 2019, there were 234,000 newly diagnosed cases of thyroid cancer and over 45,000 deaths attributed to the disease. In terms of global mortality, the ranking of thyroid cancer rose from 107th to 95th between 1990 and 2019 [1]. Thyroid cancer is the fastest-increasing of all cancers, with new diagnoses increasing rapidly around the world [4]. Though most cases have a favorable prognosis, thyroid cancer presents a heavy burden of follow-up care [4]. 

In this study, we sought to identify thyroid cancer time trends relating to incidence, mortality, and years lived with disability among the population of Saudi Arabia between 1990 and 2019. We noted a significant increase in the incidence rate of thyroid cancer in the Saudi population over the past 30 years, a finding in agreement with previous studies from around the world [9]. Thyroid cancer is a relatively rare cause of cancer death [9]. In our study period, we observed a modest increase in thyroid cancer mortality among women, but a more substantial increase among men. By 2019, the numbers of deaths among females and males had become approximately equal.

Over the 30-year study period, the incidence rate in females increased by a factor of approximately 15; in males, the incidence rose by a factor of 22. In 1990, the female proportion of Saudi thyroid cancer cases was 76.5%; by 2019, this proportion had fallen to 68.7%. The male proportion correspondingly rose from 23.5% to 31.3% in the same period. This increase in the male proportion of Saudi thyroid cancer cases has been paralleled worldwide [4], but the reason for this phenomenon is not fully clear. It might be attributed to higher exposure to known risk factors for thyroid cancer, such as exposure to radiation, obesity, and smoking. The global increase in thyroid cancer incidence might also be explained by improvements in diagnostic criteria and techniques, as well as the more frequent follow-up of incidental findings [10]. Another possible cause of increasing thyroid cancer in Saudi Arabia is a family history of the disease. The authors of reported a correlation between thyroid cancer diagnosis and previous family incidence among the Saudi population [11].

Exposure to industrial chemicals is another risk factor in thyroid cancer [12]. In addition, several studies have associated increased thyroid cancer risk with the drinking of contaminated water [13]. There is strong evidence linking thyroid disruption effects to exposure to pesticides including dichlorodiphenyltrichloroethane (DDT), hexachlorobenzene (HCB), and fungicides. Other chemicals including alachlor, 2,4-dichlorophenoxyacetic acid, aldrin, chlordane, DDT, lindane, and parathion have been found to cause hypothyroidism [14]. The structural similarity of some pesticides to the thyroid hormones can cause disruption to the thyroid function through binding of these chemicals to thyroid transporter proteins and receptors [15]. In addition, several studies have linked thyroid cancer to exposure to certain solvents and metals that have been found to affect thyroid gland homeostasis in vivo. Other studies have identified carcinogenic properties in several industrial solvents [16,17].

Tamam et al. showed that multiple (>10) dental X-ray exposures were associated with a significantly increased cancer risk [12]. They also found an increased risk of thyroid cancer after frequent diagnostic X-rays, particularly those of the head, neck, and upper back/chest region [12]. It is well known that frequent and prolonged exposure to X-rays triggers genetic alterations and causes instability in specific genes [12,18,19]. Other factors associated with thyroid cancer include smoking, a low dietary intake of iodine, high BMI, and a changing lifestyle [4].

The increase in thyroid cancer incidence and the changing difference in the male-to- female ratio suggests that occupational factors may be involved. Radiation exposure is strongly related to increased incidence of thyroid cancer. Many studies have illustrated the relationship between thyroid cancer and both direct and indirect exposure to X-rays [15], ionizing radiation, exposure to radiotherapy in early childhood, and fallout from an atomic bomb [15]. The authors of found a strong link between exposure to ionizing radiation and increased thyroid cancer cases in adult females [17].

Researchers have identified some limitations in studies of the effects of exposure to chemicals, metals [20], and industrial solvents [21,22] upon the development of thyroid cancer. These limitations include a lack of crude exposure assessments and small sample sizes. Such studies have also failed to consider possible factors such as work-based hygiene effects, biomarker-based exposure assessment, or a combination of both. The subtype of thyroid cancer most common in the exposed population needs to be stated clearly [14]. Some studies of the effects of exposure to industrial chemicals used in agriculture involved variable exposure assessment [14]. The exposure assessment in such studies has either been generally broad or has specified direct exposure to a named pesticide, fungicide, and/or insecticide. Other studies have included broad population groups based on occupation and general exposure to chemicals during work [23,24,25].

In Saudi Arabia, a career in radiology is rarely pursued by women. Several studies have pointed out low levels of interest in a radiology career among female high school students [26,27,28,29,30]. This is consistent with findings from other countries that attributed low female interest in a radiology career to future pregnancy considerations [31]. Presently, there is no publication that gives definitive numbers of male and female workers in Saudi agriculture and farming. However, based on reports in the trading economics database, we can state that the percentage of female workers in the agriculture industry is very low. In light of these findings, we can suggest that occupational exposure to thyroid cancer risk is an influencing factor behind the increased incidence of the disease among Saudi men. Such increased exposure to occupational factors such as radiation and industrial chemicals in Saudi Arabia may also explain the increased male mortality rate and the greater occurrence of aggressive subtypes among diagnosed men.

According to the Saudi Arabian National Cancer Registry, in 2017, the percentage of regional and distant disease at diagnosis was higher than 40% in male patients, compared with 28% among females. Similarly, a retrospective study of Saudi cases between 2000 and 2010 found that 53% of all male cases exhibited regional and distant metastasis at diagnosis, compared with just 22% of female cases [32]. According to data from the Saudi National Cancer Registry published in 2017, 3% of male thyroid cancer cases involved medullary carcinoma, an aggressive subtype, compared with 0.8% of female cases. Hussain et al., 2013, also found a higher incidence of medullary carcinoma in males than females in Saudi Arabia. They found that 4.41% of male thyroid cancer cases between 2000 and 2010 were diagnosed with medullary carcinoma, compared with 1.6% among females [33]. During the same period, they also found a higher percentage of anaplastic thyroid cancer, a highly aggressive subtype, in males (2.57%) compared with females (1.09%) over the same study period [33]. Taken together, these findings suggest that aggressive phenotypes are more likely to be found in males diagnosed with thyroid cancer in Saudi Arabia, and this helps to explain the phenomenon of increasing male thyroid cancer deaths.

In this study, we analyzed thyroid cancer mortality and incidence trends in Saudi Arabia from 1990 to 2019 and concluded that the death rate in the country is increasing. This is the first study to identify an increasing trend of deaths from thyroid cancer among Saudi men. The increase in the male thyroid cancer death rate is higher than that of females over the same study period. Overall, thyroid cancer cases increased by 169%, mortality by 87%, and DALYs by 75%. Similarly, the age-standardized incidence rate (ASIR) also exhibited an upward trend over the same period [13].

We found a steady increase in the years of life lost (YLLs) until the 2000s. After this, YLLs progressively increased among men and achieved parity with women by 2019. This might be explained in two ways: by increasing numbers of thyroid cancer cases in males or by increased mortality. Our findings are supported by a previous worldwide study on patients diagnosed with thyroid cancer, which assessed data of incidence, deaths, DALYs, and age-standardized parameters relating to thyroid cancer in 21 regions and 195 countries over the period from 1990 to 2017 [13]. The authors of this study found that incidence was greater among females than males, especially in South Korea. They also found a declining male ASIR, especially in Kazakhstan [13], while mortality was highest in Ethiopia in 1990, but highest in the Philippines in 2017 [13].

The current study’s limitations concern its retrospective design and inability to determine if there were regional variations in the incidence and mortality of thyroid cancer in Saudi Arabia. Given these data-type constraints, further investigations into thyroid cancer stratified by region, histologic characteristics, grade, and risk factors are needed in the future.

## 5. Conclusions

Thyroid cancer is the most common endocrine tumor worldwide and typically presents a moderate risk of death. The median age of thyroid cancer diagnosis in Saudi Arabia is 35–39 years. The median mortality age is 45–49 years, compared with a global figure of 60–64 years. These findings reflect increased cases of advanced thyroid cancer types such as medullary carcinoma. In addition, the lower median age of death from thyroid cancer might indicate a poor response to treatment. More effort is needed to determine the causes of increasing thyroid cancer cases in younger age groups of the Saudi population compared with the rest of the world. Future studies might consider treatment response, diagnosis strategy, and awareness of the disease in Saudi Arabia.

## Figures and Tables

**Table 1:** Thyroid cancer prevalence, incidence, deaths, YLLs, YLDs, and DALYs, expressed as totals and as age-standardized rates for females and males in 2019, with percentage changes between 1990 and 2019.

Gender	Prevalence		Incidence		Deaths		YLLs		YLDs		DALYs	
	Numbers (millions)	Rate (per 100,000)	Numbers (millions)	Rate (per 100,000)	Numbers (millions)	Rate (per 100,000)	Numbers (millions)	Rate (per 100,000)	Numbers (millions)	Rate (per 100,000)	Numbers (millions)	Rate (per 100,000)
**In 2019**												
Both sexes	20142 (13,443–28,317)	56.4 (37.6–79.2)	2259 (1528–3157)	6.3 (4.3–8.8)	121 (93–154)	0.3 (0.3–0.4)	4233 (3127–5495)	11.8 (8.8–15.4)	1146 (642–1858)	3.2 (1.8–5.2)	5380 (3862–7281)	15.1 (10.8–20.4)
Females	13,959 (7901–20,675)	93.8 (53.1–140)	1551 (886–2292)	10.4 (6–15.4)	60 (44–81)	0.4 (0.3–0.5)	2123 (1437–2953)	14.3 (9.7–19.9)	782 (365–1323)	5.3 (2.6–8.9)	2905 (1896- 4176)	19.5 (12.7–28.1)
Males	6183 (4272–9232)	29.6 (20.5–44.3)	708 (491–1047)	3.4 (2.4–5)	61 (46–79)	0.3 (0.2–0.4)	2110 (1549–2833)	10.1 (7.4–13.6)	365 (209–618)	1.8 (1.0–3.0)	2475 (1801–3381)	11.9 (8.6–16.2)
**Percent of Change 1990–2019**												
Both sexes	17.4% (9.4–30.4)	7.3% (3.6–13.1)	15.6% (8.2–27)	6.5% (3.1–11.6)	3.2% (1.5–5.2)	0.9% (0.1–1.8)	3.7% (1.8–6.2)	1.1% (0.3–2.2)	16.4% (8.3–28.9)	6.8% (3.2–12.4)	4.6% (2.2–7.7)	1.5% (0.4–2.9)
Females	15.3% (6.7–28.6)	6.7% (2.7–13.1)	14% (6.2–26.1)	6.1% (2.4–11.9)	2.2% (0.6–4.1)	0.5% (0.2–1.4)	2.6% (0.8–5)	0.7% (0.1–1.9)	14.5% (5.9–28)	6.4% (2.3–12.8)	3.6% (1.3–6.6)	1.2% (0.1–2.6)
Males	25.1% (14.3–44.5)	10.2% (5.6–18.6)	20.9% (12.1–36.4)	8.4% (4.6–15.1)	5% (2.9–8.4)	1.6% (0.7–3)	5.7% (3.2–9.7)	1.9% (0.8–3.6)	22.4% (12.5–40.6)	9.1% (4.8–16.9)	6.5% (3.6–11)	2.2% (1–4.2)

**Table 2:** Morphological distribution of thyroid cancer among the Saudi population, 2017.

Morphology	Male		Female	
	No.	%	No.	%
Papillary carcinoma, NOS	132	56.4	488	55.9
Papillary carcinoma, follicular variant	24	10.3	144	16.5
Papillary microcarcinoma	27	11.5	122	14.0
Follicular carcinoma, minimally invasive	7	3.0	21	2.4
Papillary carcinoma, encapsulated	6	2.6	21	2.4
Oxyphilic adenocarcinoma	4	1.7	20	2.3
Follicular adenocarcinoma, NOS	6	2.6	16	1.8
Papillary carcinoma, columnar cell	5	2.1	12	1.4
Medullary carcinoma, NOS	7	3.0	7	0.8
Others	16	6.8	22	2.5
Total	234	100	873	100

Data adapted from the National Cancer Registry of Saudi Arabia.

## Data Availability

Not applicable.
